# Supplementary material for: Transcriptome survey and expression analysis reveals the adaptive mechanism of ’Yulu Xiang’ Pear in response to long-term drought stress
Source: PLoS One. 2021 Feb 5;16(2):e0246070. doi: 10.1371/journal.pone.0246070 (PMC7864722; doi:10.1371/journal.pone.0246070)
Supplement: Attachment — Submitted filename: responses_to_technical_check_results.doc [file pone.0246070.s001.doc]

**Responses to technical check results**

Journal Name: **PlOS ONE**

Manuscript Title:

**Transcriptome survey and expression analysis reveals effects and metabolic pathways of 'Yulu Xiang' Pear in response to long-term drought stress**

Dear Editor,

Thank you for your useful comments and suggestions on the language and structure of our manuscript. We revised the whole manuscript carefully to avoid language errors. In addition, we asked several colleagues who are skilled authors to check the English. We believe that the language is now acceptable for the review process.

The manuscript has been resubmitted to your journal. We look forward to your positive response.
