# Supplementary material for: Transcriptome survey and expression analysis reveals the adaptive mechanism of ’Yulu Xiang’ Pear in response to long-term drought stress
Source: PLoS One. 2021 Feb 5;16(2):e0246070. doi: 10.1371/journal.pone.0246070 (PMC7864722; doi:10.1371/journal.pone.0246070)
Supplement: Attachment — Submitted filename: Responses to reviewers.docx [file pone.0246070.s002.docx]

**Responses to Reviewers**

Dear Editor

We want to thank you and 2 reviewers for your useful comments and suggestions on our manuscript. We have modified the manuscript, added more information accordingly, and detailed corrections are listed below point by point:

Journal requirements:

1) Please ensure that your manuscript meets PLOS ONE's style requirements, including those for file naming. The PLOS ONE style templates can be found at

https://journals.plos.org/plosone/s/file?id=wjVg/PLOSOne_formatting_sample_main_body.pdf and

https://journals.plos.org/plosone/s/file?id=ba62/PLOSOne_formatting_sample_title_authors_affiliations.pdf
AUTHOR RESPONSE:

Thank you for your suggestions, and modifications including format among others of Abstract, Introduction, Materials and Methods have been made according to PLOS ONE's style requirements.

2）Please include your tables as part of your main manuscript and remove the individual files. Please note that supplementary tables should be uploaded as separate "supporting information" files.

AUTHOR RESPONSE:

Thanks for your comments. The tables and have been included as part of manuscript. The supplementary files have been deleted, and is moved to the paper as as part of manuscript. Thanks again.

REVIEWER 1:

Reviewer #1: The article “Transcriptome survey and expression analysis reveals effects and metabolic pathways of 'Yulu Xiang' Pear in response to long-term drought stress” measures some physiological indicator and has a RNA-Seq on the drought stress pears. I have some suggestions as follows.

1. Combine figure1-figure5 into one figure.

AUTHOR RESPONSE:

Thanks for your useful suggests. The Figure 1-5 have been combined into one figure. The corresponding information in results are modified, and the new figure can be found in attached files . Thanks again.

2. Please present the useful information of RNA-Seq in the main text.

AUTHOR RESPONSE:

Thanks for your comments. The information about RNA-Seq have been rewritten , and modified information could be found in the results of new manuscript, which have been noted in the Results with red text. Thanks again for your suggestions.

3. How many repetitions of the RNA-Seq and other data.

AUTHOR RESPONSE:

We are sorry for our mistakes. There were six replicates for each treatment used for the RNA-Seq, and the experiments about fruit quality, photosynthetic indexes, antioxidant system were repeated three times. The above content have been added in the new paper. Thanks again for your comments.

4. Please describe a model in the end of the text based on your data.

AUTHOR RESPONSE:

Thanks for your suggestions. Basing on the results of biochemical indexes and transcriptome sequencing, we made a description containing words and model figure about the adaptive mechanism of 'Yulu Xiang' Pear in response to long-term drought stress. The added contents could be found in paper in the conclusion of paper. Thanks again.

5. The results are simple, rewrite it.

AUTHOR RESPONSE:

Thanks for your comments. Indeed, the results are too simple to describe our works. Therefor, Results are rewritten and modified, and more contents are added into the Results, Discussion, and Conclusion. Thanks again for your useful suggestions.

REVIEWER 2:

1. The authors had mention the water stress condition created for the 12 months by withholding irrigation in the drought stress group, however, there is no evidence shown in the result to monitored the stress level for this large period. They could have to monitor the relative water content, chlorophyll content, cell membrane stability periodically.

AUTHOR RESPONSE:

Thanks a lot for your suggestions. Undoubtedly, your comments are absolutely correct. The detection of water content in soil or other indexes should be carried out every three or four months, which is more reasonable and reliable. However, we just take one test after 12 months, and it is an irreparable mistakes in experimental design. We will take your suggestion in future study, and make our effort to avoid similar mistakes. Thanks again for your useful suggestions.

2. The authors have mentioned the soil relative water content in Pear trees cultivation was achieved 80% and 30% in control and drought stress groups respectively, but it is not cleared that at what stage the said moisture levels were achieved and for how much period these levels were maintained to induce the stress in pear trees.

AUTHOR RESPONSE:

We are so sorry for the missing description in Test Design. In fact, the pear tress in control group were given reasonable irrigation as follows: Pear trees were watered for the first time after fertilization in autumn (October 10, 2017) , and the ground was also covered with plastic film to save water in winter. Another three watering was carried out on March 5^th^( 2018), May 15^th^ (2018), and July 1^st^ (2018), respectively. For drought treatment groups, there was no water spray in the orchard.

Based on the above description, we think that the long term drought stress lasted for one year. However, the detection of soil relative water content obtained on October 21 (2018) could not fully prove the drought stress on tress during the whole year. As the response to your first question, more detection about the soil relative water content in different time is more reasonable. This is still an irreparable mistakes during the experiments. Thanks again for your comments.

3. The sources for the software packages and databases used for sequence analysis and annotation were not mentioned in the manuscript. The sources/links should be cited or acknowledged in the manuscript.

AUTHOR RESPONSE:

Thanks for your suggestion. We really appreciate all the help from the software and databases, and more related information are added in the Method. Thanks again for your suggestion.

4. The functions of the selected genes for the quantitative real-time PCR analysis were not discussed in the manuscript, on what basis the gene was selected for validation? The candidate genes for drought stress in Pear plant should be discussed in the discussion part of the manuscript correlating with the antioxidant enzymes assay, fruit soluble solids, and photosynthetic characters performed in the current research work.

Proline content is expected to be increase during drought stress conditions but in the current study, it is lower in drought stress than the control group. Please clarify it in the discussion with reference as it is a contrasting result.

The antioxidant enzymes SOD, CAT, POD activities reported decreased under drought stress as compared to the control group. At initial stress, these antioxidant enzyme levels should increase but might be because of the long-term moisture stress condition these enzyme activities are reduced. Please give references for the same. Also, the authors why not monitored the enzyme activity during stress conditions periodically so that the stage at which the activity is at its pick level could have detected.

The changes in fruit soluble solid content and quality of fruit were checked in the current work, have there any impact of drought stress on fruit-bearing and yield were not mentioned, the yield and production-related data of the control and drought stress groups should be included in the results as it directly indicates the stress impact on the production/yield from that plant.

The discussion part of the manuscript has to improve by including some additional references.

AUTHOR RESPONSE:

According to your suggestion, more information about the functions of the selected genes and relation between candidate genes with antioxidant enzymes assay, fruit soluble solids, and photosynthetic characters are added in the part of Discussion and conclusion. And, more related literatures are cited. Thanks again for your suggestions, and detailed information can be found in newly modified articles.

5. Besides these points, there are some minor typo and grammatical errors are there which need to be corrected.

L 172 – Add “and” in-between two values as it creating the confusions.

L 174 - L 176- Modify the sentence for more clarity.

L 210 - Is the author wants to say “for further study to invest ‘investigate’ the functions of drought stress-responsive genes”? Correct the sentence.

L 231 – L 232- Reference is missing for the statement.

L 258 - L 260- Add reference for the sentence.

Fig. 6 & Fig. 7- The labels of the X-axis are difficult to read, use good quality graphical image.

AUTHOR RESPONSE:

Thank your for your suggestion and comments. The incorrect or incomprehensible sentences have been modified, and relative literature is cited. The figure is replaced with another good quality graphical image, and more information could be found in newly modified articles. Thanks again for your suggestions
